# Effect of Inspiratory Muscle Training on Diaphragm and Abdominal Wall Muscle Thickness with Fatty Liver Density in Elderly Women: A Randomized Controlled Trial

**DOI:** 10.3390/medicina61101784

**Published:** 2025-10-02

**Authors:** Eda Gökçelik, Coşkun Yılmaz, Cemallettin Budak, Hakan Hüseyin Soylu, Serdar Bayrakdaroğlu, Halil İbrahim Ceylan, Raul Ioan Muntean, Hamza Küçük, Levent Ceylan

**Affiliations:** 1 Faculty of Sport Sciences, Istanbul Aydın University, Istanbul 34295, Türkiye; edagokcelik@aydin.edu.tr; 2 Kelkit Aydın Doğan Vocational School, Gümüşhane University, Gümüşhane 29600, Türkiye; coskun.yilmaz@gumushane.edu.tr; 3 Faculty of Sport Sciences, Erzincan Binali Yıldırım University, Erzincan 24100, Türkiye; cemalettin.budak@erzincan.edu.tr; 4 Radiology Department, Kelkit District State Hospital, Gumushane Provincial Health Directorate, Gümüşhane 29600, Türkiye; huseyin.soylu.91@gmail.com; 5 Faculty of Sports Sciences, Gümüşhane University, Gümüşhane 29100, Türkiye; bayrakdaroglu85@gmail.com; 6 Faculty of Sports Sciences, Atatürk University, Erzurum 25100, Türkiye; 7 Faculty of Law and Social Sciences, University “1 Decembrie 1918” of Alba Iulia, 510009 Alba Iulia, Romania; 8 Yaşar Doğu Faculty of Sports Sciences, Ondokuz Mayıs University, Samsun 55100, Türkiye; hamza.kucuk@omu.edu.tr; 9 Faculty of Sport Sciences, Hitit University, Çorum 19030, Türkiye; leventceylan17@hotmail.com

**Keywords:** elderly women, health, abdominal muscle, exercise, diaphragm, aging, inspiratory muscle training

## Abstract

*Background and Objectives:* Post-menopausal estrogen decline is considered a contributing factor to sarcopenia, and inspiratory muscle training (IMT) may provide benefits in this demographic. This study examined the impact of a four-week IMT program on diaphragm thickness, abdominal wall muscle thickness (AWMT; transversus abdominis, internal oblique, and external oblique), and liver fat percentage in healthy elderly women. *Materials and Methods:* Twenty-six women aged 60–80 years were randomly assigned to an IMT group (*n* = 13) or a control group (*n* = 13). The IMT group used the PowerBreathe^®^ Classic device at 40% of maximal inspiratory pressure (MIP), with weekly increments of 10%. Training was performed twice daily, five days per week, with 30 breathing cycles per session (60 per day). The control group maintained their usual routines. AWMT, diaphragm thickness (DT), and fatty liver density (FLD) were measured by a radiologist before and after the intervention. *Results*: After four weeks, the IMT group showed significant improvements in all parameters compared to controls. Mid-diaphragm thickness (MDT) increased by 11.44% (effect size (ES) = 0.358, *p* < 0.001) versus 0.76% in controls (*p* = 0.271). Posterior diaphragm thickness (PDT) improved by 7.48% (ES = 0.282, *p* < 0.001) versus 0.38% (*p* = 0.564). Right AWMT increased by 12.7% (ES = 0.492, *p* < 0.001) compared to 0.10% (*p* = 0.872), and left AWMT increased by 9.93% (ES = 0.395, *p* < 0.001) versus 2.64% (*p* = 0.014). FLD improved by 11.79% (ES = 0.959, *p* < 0.001) in the IMT group, while the control group showed no meaningful change (−0.13%, *p* = 0.847). *Conclusions:* A short-term IMT protocol significantly enhanced diaphragm and AWMT and reduced liver fat in elderly women. These findings support the use of IMT as a simple, non-invasive intervention to preserve musculoskeletal and metabolic health in aging populations.

## 1. Introduction

The aging process leads to various impairments in multiple physiological systems, including the musculoskeletal and respiratory systems. One of the primary consequences of this process is the decline in overall muscle strength, which directly affects an individual’s functional capacity and ability to perform daily living activities. Although this reduction in muscle mass and strength is not directly associated with any specific disease, it becomes more pronounced in elderly adults with health conditions that limit mobility [1]. Respiratory muscle weakness alone is considered a significant limiting factor for physical fitness and may lead to clinical outcomes, including impaired lung function, reduced muscle strength, and dyspnea [2].

Previous studies have reported that physical exercise provides a wide range of benefits for elderly adults, including improvements in functional capacity, reductions in infection rates, enhanced cardiovascular fitness, improved muscle fiber quality, and overall enhancements in quality of life [3,4]. Aerobic exercise has been shown to exert systemic benefits, including improvements in respiratory muscle strength. Moreover, regular physical activity induces both local and systemic anti-inflammatory responses, offering protective effects against age-related chronic inflammatory pathologies [5,6].

In this context, improving respiratory function through targeted interventions is vital for maintaining independence and decreasing healthcare burdens in aging populations. In recent years, IMT has gained increasing attention due to its dual role in enhancing athletic performance and improving functional capacity in elderly adults [7,8,9,10,11]. IMT is a resistance training approach involving moderate-intensity respiratory loading, typically at 40–60% of maximal inspiratory pressure. It is performed over short durations while yielding significant improvements in muscle strength and endurance [12]. Home-based protocols generally consist of breathing sessions conducted twice daily, five to seven days per week [13].

The practicality and safety of these home-based programs make IMT particularly suitable for elderly populations, including those with mobility restrictions. IMT has been reported to lower blood pressure in elderly adults with systolic hypertension [14], improve cardiac autonomic function [1], reduce sympathetic overactivity, and enhance vagal tone at rest in individuals with obstructive sleep apnea [15,16,17]. Furthermore, IMT has been identified as a non-pharmacological intervention that enhances diaphragm function, supports postural balance, increases trunk stability, facilitates venous return, and ultimately reduces the risk of falls [18,19,20].

With advancing age, several age-related changes occur in the respiratory system, including reduced chest wall compliance, decreased elastic recoil of the lung parenchyma, diminished respiratory muscle strength, and a blunted response to gas exchange abnormalities [11]. These alterations are associated with decreased exercise capacity, impaired walking performance, and reduced quality of life in elderly adults [21]. The decline in respiratory muscle function renders elderly individuals more vulnerable to disease and disability; therefore, strengthening these muscle groups is crucial for reducing the risks of morbidity and mortality [22]. Despite this, respiratory-specific exercise strategies, such as IMT, remain underutilized in geriatric health promotion efforts.

Although peripheral muscle exercises may yield partial gains in respiratory muscle function, more pronounced improvements have been observed when these exercises are supported with inspiratory muscle-specific training [23]. In the literature, the diaphragm has been reported to play an active role not only in respiratory function but also in controlling postural balance. By increasing intra-abdominal pressure, the diaphragm contributes to spinal stabilization and, in coordination with the abdominal muscles, supports balance [19,24]. In this context, ultrasound imaging can be used to measure abdominal muscle thickness, and a positive correlation between muscle thickness and strength has been reported [25,26].

Previous studies have primarily focused on the effects of IMT on respiratory performance, diaphragm mobility, and functional capacity in elderly populations [27,28,29]. While these studies consistently reported improvements in inspiratory pressures and diaphragm thickness, they did not comprehensively address changes in AWMT or explore systemic outcomes such as hepatic steatosis. Furthermore, most available research has examined either functional or structural outcomes separately, leaving a gap in understanding the dual effects of IMT on both musculoskeletal and metabolic health indicators. Our study differs from prior work in that it evaluates not only diaphragm thickness and AWMT but also liver fat density (FLD) using computed tomography in elderly women. This combined approach offers novel insights into how a short-term IMT intervention may impact both muscle structure and metabolic health. To address this gap, the present study aimed to investigate the effects of a four-week IMT intervention on diaphragm thickness and AWMT, as well as FLD, in this population. We hypothesized that IMT would lead to significant improvements in these outcomes, thereby supporting its role as a comprehensive strategy to preserve both functional capacity and metabolic health in aging women.

## 2. Materials and Methods

### 2.1. Participants

This study was conducted as a parallel-group, pretest-posttest randomized controlled trial following the CONSORT guidelines. Before participation, all individuals received detailed information about the study and provided written informed consent following the ethical principles outlined in the Declaration of Helsinki. The study was designed in accordance with the principles of the Declaration of Helsinki and was approved by the Scientific Research Ethics Committee of Istanbul Aydın University (date: 20 February 2025, number 2025/2). The sample size was calculated using G*Power software (version 3.1.9.2) based on diaphragm thickness (DT) values reported in previous studies [30], with an alpha error of 0.05, a beta error of 0.2, and a medium effect size (f = 0.25 or partial eta squared = 0.06). Considering the study design, a dropout rate of approximately 50% was anticipated. Accordingly, a sample size of 20 participants (n = 10 per group) was initially determined. However, to minimize potential issues, it was decided to include a total of 30 participants by adding 50% more participants to each group. Ultimately, four participants were excluded because they did not meet the inclusion criteria, and the study was completed with 26 participants. To determine which group the subjects forming the sample would be included in, the numbers from 1 to 26 were randomly assigned to two groups through a computerized program (https://www.randomizer.org/). The two groups participating in the study consisted of healthy elderly women residing in a special care home, who were retired from their working lives and had similar levels of physical activity.

The inclusion criteria included women aged 60–80 years (post-menopausal), who were independent in activities of daily living, demonstrated adequate cognitive function to follow instructions, had no musculoskeletal injury within the previous six months, were non-smokers, and had routine thoracic or abdominal CT images obtained for clinical indications and used in this study. The exclusion criteria included having uncontrolled cardiovascular or pulmonary disease such as heart failure or COPD/asthma exacerbation, uncontrolled hypertension, diagnosed chronic liver disease, acute respiratory infection within the last four weeks, participation in structured respiratory or resistance training within the previous six months, recent thoracoabdominal surgery or hernia, use of medications known to affect muscle or liver metabolism such as systemic corticosteroids, anabolic agents, or hepatotoxic drugs, severe orthopedic or neurological conditions limiting mobility, or refusal to participate.

### 2.2. Experimental Design

**Figure 1:**
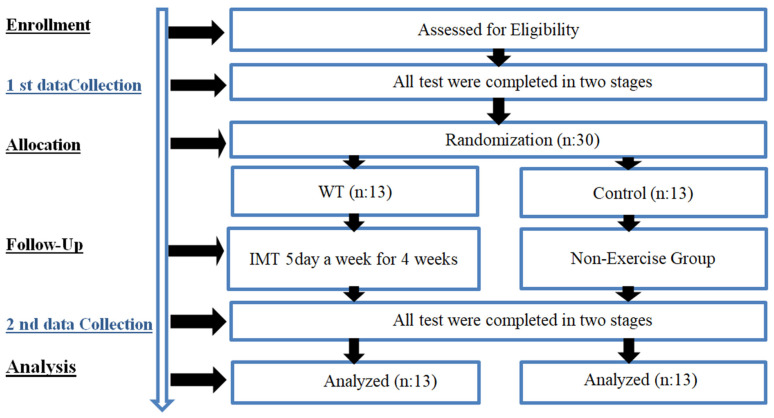
Experimental design.

Women aged 60–80 were invited to attend the laboratory on three separate occasions. During the initial visit, the experimental procedures were introduced and tested. Before the experiment began, each participant received a comprehensive explanation of the IMT procedure. Each participant underwent a week-long trial period, during which an experienced physiotherapist closely monitored them. The trial period involved the use of a customized respiratory muscle training device tailored to meet the specific requirements of each participant. During the second visit, a series of pre-training measurements was taken and recorded. The tests included evaluations of AWMT, diaphragm thickness (DT), and FLD, all of which were conducted under the supervision of a radiologist. After the four-week IMT program, final measurements were obtained during the final visit (Figure 1).

### 2.3. Body Composition Measurement

The height of participants was measured to the nearest 0.1 cm using a standard height meter (Seca 769, Seca, Hamburg, Germany) while standing barefoot against a wall. Body weight was measured to the nearest 0.1 kg using a digital scale (Beurer, model GS27, Ulm, Germany). Participants’ body weight was measured in kilograms (kg) without shoes and while wearing shorts and a T-shirt to minimize the influence on the results.

When examining the averages of the participants in the study, the IMT group had an age of 67.69 ± 9.25 years, a height of 159.5 ± 4.3 cm, and a weight of 70.77 ± 3.65 kg. For the control group, the mean age was 68 ± 8.40 years, height was 157.85 ± 3.05 cm, and weight was 69.46 ± 2.22 kg (Table 1).

### 2.4. Liver Density Analysis

In this study, CT images were preferred because participants had already undergone thoracic or abdominal CT scans as part of their routine clinical care. No additional imaging was performed for research purposes. Thus, existing clinical data were utilized, avoiding extra radiation exposure. Imaging was performed using a Siemens Somatom Definition AS 128 CT scanner (Siemens Healthcare, Erlangen, Germany) during the inspiratory phase after standardized breathing instructions [31]. Liver density was measured from a homogeneous parenchymal region without visible lesions in segment VIII adjacent to the middle hepatic vein. At the same time, the thicknesses of the diaphragm and abdominal muscles were assessed at anatomically standardized sites. The device automatically calculated and recorded liver fat content. A liver fat value of less than 33 indicated increased hepatic steatosis [32].

### 2.5. Diaphragm Thickness (DT) Measurement

Diaphragm muscle thickness measurements were obtained from computed tomography images taken for clinical reasons. The GE Revo Evo Cardiac device (Revolution EVO, Revolution Maxima, Revolution Frontier, Revolution HD, Revolution CT, GE Healthcare, Milwaukee, WI, USA) was used for imaging. A radiologist with expertise in thoracic computed tomography performed the measurements of diaphragm muscle thickness. During the imaging procedure, the subjects were in a supine position during the deep inspiration phase. Thickness measurements were obtained at the level of the upper pole of the right kidney. The measurements were taken perpendicular to the diaphragm axis, from the midline, and the posterior line of the vertebral body. At this juncture, the diaphragm muscle was visualized and its thickness was measured.

### 2.6. Abdominal Wall Muscle Thickness Measurement

The present study utilized data obtained from participants aged 60 and above who underwent chest or abdominal CT scans for various clinical indications. Imaging was performed using a computed tomography device, model Revo Evo of General Electric (Waukesha, WI, USA). Before undergoing imaging procedures, the participants were instructed to perform breathing exercises. On the day of imaging, participants were positioned supine on the device table. Following the completion of device settings, participants were asked to take a deep breath and hold it immediately before imaging commenced [31]. The images under consideration were obtained during the inspiration phase. In this study, AWMT was defined as the thickness of the muscular layers of the abdominal wall (transversus abdominis, internal oblique, and external oblique), excluding subcutaneous fat and other non-contractile tissues. For simplicity, this variable is hereafter referred to as AWMT. Measurements were taken at the level of the upper iliac wings, in a plane perpendicular to the muscle plane at the level of the anterior line of the vertebral body.

### 2.7. Inspiratory Muscle Training (IMT)

IMT was performed using the POWERbreathe^®^ device (POWER^®^ Breathe Classic, IMT Technologies Ltd., Birmingham, UK). The POWERbreathe^®^ device is a pressure-threshold IMT device that provides resistance during inhalation. The user breathes in through a mouthpiece against an adjustable spring-loaded valve, which opens only when the inspiratory pressure exceeds the set threshold, thereby strengthening the inspiratory muscles over time [33]. Following a one-week familiarization period, the IMT protocol was performed twice daily (morning and evening) for five consecutive days per week over four weeks. Each session consisted of 30 breathing cycles, totaling 60 cycles per day. Before training, the device resistance was calibrated to 40% of each participant’s maximal inspiratory pressure (MIP), as reported by Çelikel et al. [34] to promote increases in muscle size and thickness. MIP was reassessed weekly, and resistance was increased by 10%; progression was paused if participants experienced excessive strain. All participants were able to complete the progressive protocol without interruption. Importantly, all IMT sessions were supervised by a licensed physiotherapist with over five years of clinical experience in respiratory rehabilitation, who provided detailed instructions on the correct use of the POWERbreathe^®^ device, demonstrated proper breathing techniques, and corrected participant errors during the initial sessions. Morning IMT sessions were scheduled between 8:00 and 10:00 a.m., while evening sessions took place between 5:00 and 8:00 p.m.

### 2.8. Statistical Analysis

Statistical analyses were performed using SPSS (Version 21.0 for Windows, Chicago, IL, USA), with a statistical significance level set at 0.05. The Shapiro—Wilk normality test was performed to determine the normality of the sample. The pre-test and post-test differences were determined using a Paired Samples t-test and One-Way Analysis of variance (ANOVA), with post-test and pre-test difference values used to determine between-group differences. Additionally, the effect size in the comparison of paired groups was calculated using Hedges’ g [35]. It was also interpreted as follows: 0–0.19 insignificant, 0.20–0.59 small, 0.6–1.19 medium, 1.20–1.99 large, and ≥2.00 very large.

## 3. Results

When comparing pre- and post-training MDT values, the IMT group showed a significant increase (ES = 0.358; 11.44%, *p* < 0.001) compared to the control group (ES = 0.030; 0.76%, *p* = 0.271), with an overall significance of *p* < 0.001. For PDT, a more pronounced increase was observed in the IMT group (ES = 0.282; 7.48%, *p* < 0.001) compared to the control group (ES = 0.012; 0.38%, *p* = 0.564), with overall significance *p* < 0.001. Regarding right abdominal wall muscle thickness (RAWMT), the IMT group demonstrated a greater increase (ES = 0.492; 12.7%, *p* < 0.001) compared to the control group (ES = 0.004; 0.10%, *p* = 0.872), *p* < 0.001. For left abdominal wall muscle thickness (LAWMT), the IMT group exhibited a significant increase (ES = 0.395; 9.93%, *p* < 0.001) compared to the control group (ES = 0.117; 2.64%, *p* = 0.014), with overall significance *p* < 0.001. Finally, for FLD, the IMT group showed a marked increase (ES = 0.959; 11.79%, *p* < 0.001) versus the control group (ES = 0.012; −0.13%, *p* = 0.847), *p* < 0.001 (Table 2).

## 4. Discussion

The findings of the present study demonstrate that the IMT group exhibited a 10.68% enhancement in MDT, a 7.1% improvement in PDT, a 12.6% improvement in RAWM, a 7.29% improvement in LAWM, and an 11.92% improvement in FLD compared to the control group, indicating potential positive effects of IMT, particularly on the respiratory muscles and abdominal muscle groups.

A survey of the relevant literature reveals that IMT has been shown to produce significant improvements in respiratory muscle function, diaphragm thickness, and exercise capacity. Enright et al. [28] reported that high-intensity IMT application in healthy individuals resulted in significant increases in a range of parameters. These included body composition, lung function, maximum and sustained inspiratory pressures (MIP and SMIP), diaphragm thickness (Tdi.rel and Tdi.cont), and thickening ratio (TR). These findings are consistent with the data on increased MIP and abdominal muscle thickness obtained in the present study.

Similarly, Souza et al. [27] reported that moderate-intensity IMT increased respiratory muscle strength, diaphragm thickness, and diaphragm mobility in elderly women and was effective in reducing age-related functional losses. In this study, the training group showed a 37% increase in maximal inspiratory pressure, a 13% increase in maximal expiratory pressure, an 11% increase in diaphragm thickness, and a 9% increase in mobility compared to the control group. These results support the improvements observed in parameters related to abdominal muscles, such as RAWMT and LAWMT, in our study.

In a separate study, Cader et al. [29] investigated the effects of an eight-week IMT program in elderly women. They reported significant improvements in MIP and MEP values, enhanced functional capacity as measured by the 6-minute walk test (6MWT), and better quality of life scores, as indicated by the SF-36 questionnaire. The present findings demonstrate that increases in abdominal and diaphragm muscle strength are associated with functional capacity, thereby providing support for our current study. For instance, Özdoğan et al. [36] reported significant increases in MIP, diaphragm thickness, and 6DKY distance in the training group after an eight-week IMT program in their study on individuals with sarcopenia. The rise in diaphragm thickness, measured at the functional residual capacity (FRC) level (from 1.62 ± 0.28 mm to 1.72 ± 0.28 mm), is noteworthy in demonstrating the diaphragm muscle’s adaptive capacity. Similarly, Rezkallah et al. [37] investigated the effects of IMT on diaphragmatic mobility (diaphragmatic excursion) in elderly individuals, demonstrating that IMT increased the range of motion of the diaphragm during both normal and deep breathing.

A review study by Seixas et al. [38] demonstrated that IMT significantly increased inspiratory muscle strength and diaphragm thickness in elderly adults. Similarly, a systematic review and meta-analysis by Manifield et al. [11] found that IMT has a positive effect on MIP values and functional capacity in elderly adults. Rodrigues et al. [1] demonstrated that a four-week IMT program increased heart rate variability, improved 6-minute walk test (6MWT) distance, and enhanced heart rate recovery in women aged 60–72 years. Mills et al. [39] reported that IMT led to positive changes in inspiratory muscle function and diaphragm structure in healthy elderly adults; however, it did not significantly affect variables such as systemic inflammation, exercise performance, or quality of life.

In general, studies of IMT in healthy elderly individuals have demonstrated substantial enhancements in respiratory function, balance, cardiorespiratory system recovery, quality of life, immune response, lower extremity muscle strength, muscle oxygenation, exercise capacity, and physical activity levels [1,19,21,23,40,41,42]. The findings of this study demonstrate that both diaphragm and abdominal muscle function can undergo significant improvement with IMT, a conclusion consistent with the results reported in the existing literature. It is hypothesized that IMT exerts a dual effect on muscle strengthening, with the potential to enhance both the respiratory and abdominal muscles, thereby leading to an increase in functional capacity. A review of the literature shows a lack of studies investigating the effect of IMT on FLD.

The findings of this randomized controlled trial provide preliminary evidence that a short-term IMT protocol may lead to improvements in diaphragm and AWMT, as well as liver density, in healthy elderly women. Given the non-invasive, low-cost, and easy-to-implement nature of IMT, this intervention can be incorporated into community-based health promotion programs, geriatric rehabilitation settings, and home-based exercise routines to support functional capacity and metabolic health in aging populations. Clinicians, physiotherapists, and geriatric exercise specialists could consider IMT as a supportive strategy to counteract age-related declines in respiratory and core muscles, enhance postural stability, and potentially reduce the risk of hepatic steatosis. The simplicity of using portable devices like the POWERbreathe^®^ makes daily compliance feasible, even among elderly adults with limited mobility. Ultimately, incorporating IMT into multidisciplinary healthy aging protocols may contribute to a better quality of life and help reduce healthcare burdens in elderly populations. However, larger and longer-term studies are needed to confirm these findings.

### Implications and Limitations

This study has several limitations that should be considered when interpreting the findings. First, the relatively small sample size (n = 26) reduces statistical power and limits the generalizability of the results. Additionally, the sample size was calculated based on diaphragm thickness, which may not fully represent all the study’s primary outcomes. Second, the study included only elderly women, which prevents conclusions about sex-based differences or applicability to other age groups. Third, although the IMT protocol was performed twice daily for four weeks with progressive resistance, the total duration and volume of the intervention may have been insufficient to induce significant increases in muscle thickness, potentially limiting the magnitude of structural adaptations observed. Longer-term or higher-volume training protocols may provide more robust morphological changes. Fourth, lifestyle-related confounding factors, including participants’ occupational workload, daily activity levels, and dietary habits, were not systematically monitored, which may have influenced muscle and metabolic outcomes and potentially introduced uncontrolled variability. Furthermore, the study focused on morphological outcomes (muscle thickness and liver density) and did not include functional parameters, such as inspiratory pressure, physical performance, or quality of life. Additionally, it did not assess body composition or biochemical markers of metabolic or inflammatory status. These limitations suggest that while the present findings provide preliminary evidence of the potential benefits of IMT, future research should include larger and more diverse populations, extended follow-up periods, and a broader set of functional and metabolic outcomes to better establish the role of IMT in aging populations.

## 5. Conclusions

The present study confirmed the central hypothesis that IMT improves the thickness of the diaphragm and AWMT, as well as FLD, in elderly women. Additionally, AWMT and FLD showed greater improvement in elderly women who included IMT in their routines compared to those who did not. Based on these findings, it can be concluded that IMT may have positive effects not only on local muscle structure and strength but also on systemic health indicators in elderly individuals. This suggests that adding IMT to daily activities for elderly women is advisable. Future research should investigate the physiological and biochemical mechanisms underlying these effects in greater detail.

## Figures and Tables

**Table 1:** Demographic information of the two groups.

	IMT (n:13)		Control Group (n:13)	
	X	S.D.	X	S.D.
Age (years)	67.69	9.25	68	8.40
Height (cm)	159.15	4.3	157.85	3.05
Body Weight (kg)	70.77	3.65	69.46	2.22

X; Mean, S.D.; Standard deviation.

**Table 2:** Within and Between-Group Comparison of diaphragm, AWMT, and FLD pre- and post-training in IMT and control group.

		IMT					Control Group					P_3_
		Mean	S.D.	%	E.S.	P_1_	Mean	S.D.	%	E.S.	P_2_	
MDT (mm)	Pre	2.97	0.96	11.44	0.358	*p* < 0.001	2.60	0.67	0.76	0.030	0.271	*p* < 0.001
	Post	3.31	0.94				2.62	0.65				
PDT (mm)	Pre	2.94	0.78	7.48	0.282	*p* < 0.001	2.60	0.84	0.38	0.012	0.564	*p* < 0.001
	Post	3.16	0.78				2.61	0.79				
RAWMT (mm)	Pre	17.95	4.71	12.7	0.492	*p* < 0.001	19.72	4.62	0.10	0.004	0.872	*p* < 0.001
	Post	20.23	4.55				19.70	4.59				
LAWMT (mm)	Pre	17.21	4.51	9.93	0.395	*p* < 0.001	19.63	4.47	2.64	0.117	0.014	*p* < 0.001
	Post	18.92	4.14				20.15	4.43				
FLD (HU)	Pre	60.69	8.40	11.79	0.959	*p* < 0.001	59.70	6.84	−0.13	0.012	0.847	*p* < 0.001
	Post	67.85	6.39				59.62	6.65				

MDT: mid-diaphragm muscle thickness, PDT: posterior diaphragm muscle thickness, RAWMT: right abdominal wall thickness, LAWMT: left abdominal wall thickness, FLD: fatty liver density, P_1_: IMT group significance level, P_2_: Control group significance level, P_3_: Intergroup comparison significance level, E.S.: Effect size.

## Data Availability

All datasets on which the results of an article were based were included as part of the submission.
